# French-speaking Africa and translation: From midwifery to maïeutique?

**DOI:** 10.18332/ejm/146546

**Published:** 2022-05-18

**Authors:** Annie-Hortense Atchoumi, Joeri Vermeulen, Etienne Tsou, Céline Lemay, Claire de Labrusse, Christine Morin, Yvonne Meyer

**Affiliations:** 1 Association of Midwives of Cameroon, Yaoundé, Cameroon; 2 Central African Midwifery Network, Yaoundé, Cameroon; 3 Federation of Midwifery Associations of Francophone Africa, Yaoundé, Cameroon; 4 Department Health Care, Erasmus Brussels University of Applied Sciences and Arts, Brussels, Belgium; 5 Faculty of Medicine and Pharmacy, Department of Public Health, Biostatistics and Medical Informatics Research group, Vrije Universiteit Brussel, Brussels, Belgium; 6 Health Science Faculty, Catholic University of Central Africa, Yaoundé, Cameroon; 7 Université du Québec à Trois-Rivières, Trois-Rivières, Canada; 8 HESAV School of Health Sciences, HES-SO University of Applied Sciences and Arts Western Switzerland, Lausanne, Switzerland; 9 Midwifery School, University Hospital Center of Bordeaux, Bordeaux, France

**Keywords:** midwifery education, midwifery practice, African midwives, midwifery associations, midwifery professionalization

## Abstract

**INTRODUCTION:**

In the French version of The Lancet Series (2014) midwifery has been translated as *maïeutique*. Likewise, the term *maïeuticien* has recently been introduced in some countries to name (male) midwives. This change of terminology has not been the subject of broad stakeholder consultation. The aim of this study is to explore the opinion of African midwives on the use of the terminologies pratique de *sage-femme/maïeutique* (midwifery) and *sage-femme/maïeuticien* (midwife).

**METHODS:**

A quantitative study was conducted using an online survey among members of francophone professional midwifery associations in 17 French-speaking African countries.

**RESULTS:**

From 140 invited midwives, 82 responses were received. The respondents represented 12 francophone African countries. Respondents obviously prefer the terms pratique de *sage-femme* and *sage-femme* above *maïeutique* and *maïeuticien*. The *sage-femme* is acknowledged and deeply rooted in African society. Midwifery is comprehensive, while *maïeutique* does not describe the full scope of midwifery. Though, some respondents believe that *maïeutique* has the potential to differentiate sages-femmes from other health professionals, can diminishing role ambiguity, and value midwifery practice. Respondents in favor of the term *maïeutique* are referring to the modernization of the midwifery profession and its scientific evolution.

**CONCLUSIONS:**

Internationally, midwives closely follow the developments on the linguistic subject of *maïeutique*. The results of this study may support current discussion about the evolution and modernization of terminology in the francophone community worldwide. Midwives need to be actively involved in these discussions. Nevertheless, at all times we need to be cautious not to break away from midwives’ cherished historical, social, and cultural roots.

## INTRODUCTION

Midwifery was recently defined in The Lancet Midwifery Series as the ‘skilled, knowledgeable, and compassionate care for childbearing women, newborn infants and families across the continuum throughout pre-pregnancy, pregnancy, birth, postpartum, and the early weeks of life’^1^. On the cover page of the Executive Summary for this Lancet’s Series it is stated that ‘midwifery is a vital solution to the challenges of providing high-quality maternal and newborn care for all women and newborn infants, in all countries’^2^. Therefore, expending the midwifery workforce and investing in midwifery are effective strategies to reduce maternal and neonatal mortality and improve sexual and reproductive health.

The Lancet made an important effort by publishing simultaneously, in June 2014, the Midwifery Series in both English and French. With 300 million speakers, up by nearly 10% since 2014, the French language is the fifth most spoken language in the world. Present on all 5 continents, the French language displays all the characteristics of a global language^3^. In this perspective, the French version of The Lancet Series is most welcomed, but the translation of midwifery as *maïeutique* is problematic. A study on the meaning and impact of such a translation has revealed a significant loss of meaning when using the term *maïeutique*. With its roots in ancient Greek philosophy, the term *maïeutique* focuses on aspects dealing with the philosophical, ethical and teaching strategies, which are part of the midwifery values and skills^4^. But *maïeutique* does not consider the fundamental part of the clinical field of midwifery practice and expertise^3^.

In 2020, the French National Council of Universities defined *maïeutique* as a medical discipline, in the field of perinatal and reproductive and sexual health, exercised by midwives^5^. The introduction of the term *maïeutique* was part of the claim for a body of knowledge specific to midwives, which differs from the medical disciplines of obstetrics and gynecology^6^. Nguyen, former president of the French National Conference of Maïeutics’ Teachers, demonstrated the genesis of the terminological choice of *maïeutique* in France^4^. Likewise, the term *maïeuticien* has recently been legally introduced in some countries to name (male) midwives.

However, in French, midwifery cannot be translated only in one word, which may explain the pragmatical choice of the translators. Prior to The Lancet’s translation of midwifery into *maïeutique*, the term commonly used was *pratique de sage-femme or pratique sage-femme*^6^. The change of terminology, which is supposed to define the discipline specific to midwives has not been the subject of broad stakeholder consultation. Moreover, French-speaking associations of midwives worldwide were not involved in this fundamental reflection. Some sociopolitical actors reinforce the problem. Especially in France, the Inspection Générale des Affaires Sociales, whose mission is in particular to advise the French public authorities, recommend a new degree of *praticien en maïeutique*, only accessible after a 15-year career under certain conditions of education^7^. This recommendation is not currently retained. If the French Government validates this measure, the professional course would be student in *maïeutique* during education, *sage-femme* for graduates and praticien *maïeuticien* for an executive position. This paradigm shift causes doubts whether the naming is related to the same profession or whether ultimately the naming *sage-femme* may disappear. Indeed, in 2021 the French national organization of information on education and professions (Office national d'information sur les enseignements et les professions, ONISEP), presented a report on midwifery education, named *les études de maïeutique*, (*sage-femme*). In addition, ONISEP allowed the choice of becoming a *sage-femme* or *maïeuticien*^8^.

In the French-speaking countries of sub-Saharan Africa, similar terminology problems exist. A French expert in assessment and accreditation of midwifery education programs in Ivory Coast, Mali and Chad described using established wordings as *institutions d’enseignement de la maïeutique, couverture en soins de maïeutique or soins de maïeutique de qualité*^9^. It remains unsure if the use of such terminology contributes to the improvement of the education of midwives.

Although the English word midwife is equivalent to the French term *sage-femme*, a gender issue has arisen in some national French settings and the term *sage-femme* (midwife) has become *maïeuticien* for men^6^. The term *maïeuticien* has been introduced in some African countries (Burkina-Faso, Cameroon, etc.) to name male midwives. This, despite the fact that male midwives existed long before and were commonly called *accoucheur*, birth attendants. The term *accoucheur* is nowadays still in use in some countries such as South Africa, to name male midwives^10^. In Central Africa several countries integrated male midwives in their health system. In a study conducted by Bwalya et al.^11^ regarding the perceptions of pregnant women towards male midwives, it was indicated that majority of women in Zambia (83%) accepted the care provided by male midwives with the opinion that both female and male midwives received the same training, and hence offered the same care^11^. A recent study in South Africa revealed that postpartum mothers preferred care by male midwifery students as they were viewed to be respectful, empathic, and caring. The study recommends that public awareness should be created about the availability and promote acceptability of male midwifery students in maternity units^12^.

In several African countries, the fundamental issue faced by midwives is their unclear identity buried among a multitude of health workers educated and employed as midwives who do not meet the International Definition of the Midwife of the ICM (International Confederation of Midwives)^13^. In several African countries midwifery, *la pratique de sage-femme*, is carried out by different health professionals such as obstetricians, general practitioners, nurses, registered and community midwives, community health extension workers, and often traditional birth attendants^2,14^. There is no specific regulatory body for midwives in some African countries and midwifery is not legally classified as an autonomous profession in many^4^. The midwifery profession, for example in the Democratic Republic of Congo, mirrored through the theory of a profession^15^, had not sufficiently acquired all characteristics a profession should have, namely a scientific body of knowledge, competency, an ethical code, a license to practice autonomous, and the formal recognition of society^16,17^. In addition to this, the legislation creating an order and the midwifery ethical code incorporated in some African countries the name *maïeutique* to align with the Lancet Midwifery Series. Nevertheless, *maïeutique* cannot be translated into all African local languages, which remains an obstacle to get established.

Global standards comprise two types of midwifery education: a minimum of three years for direct-entry midwifery programs, or a 1.5-year post-nursing program^18^. Varying study duration, study load, study outcomes and degrees of education are described in Africa^1^. Gradually there have been several levels of education with different qualifications. A recent study describing findings from an assessment of midwifery education in Ethiopia, Ghana, and Malawi, concluded that the various educational pathways for midwifery study reflect the view of midwifery both as a traditional occupation and an emerging profession. Global pathways to midwifery are very diverse. Global case studies indicate that there is no uniform system of routes of entry to initial preparation for midwifery, and no overall consensus regarding the optimal model for such education. Programs are available at technical, baccalaureate, and graduate degree levels^19^.

The integration of midwifery programs within degreegranting pathways, such as license Master’s and doctorate level (LMD system), recently had been introduced in Tunisia and Morocco as a reform^2^. At the same time, in several East African countries (e.g. Uganda, Kenya and Tanzania) pathways are under construction offering licensed diploma midwives the opportunity to study towards a Bachelor’s degree in midwifery^1^. It remains unclear what name is better suited for the profession within the upcoming LMD system in the French-speaking part of Africa. The aim of this study is to explore the opinion of African midwives on the use of the terminologies *pratique de sage-femme/maïeutique* (midwifery) and *sage-femme/maïeuticien* (midwife). The results of this study may support current discussion about the evolution and modernization of terminology in the francophone community worldwide.

## METHODS

### Study design

A quantitative study using an online survey was designed. The principal researcher (AHA) and a colleague (ET) both from the midwifery association of Central Africa in Cameroon constructed a questionnaire to determine:

- If the translation of midwifery is equivalent in its meaning to *maïeutique*;
- What is the impact of this translation; and
- The advantages and disadvantages of the use of both terms *sage-femme* and *maïeutique*.

Content validity of the instrument was ensured through the constructive criticism from expert midwives^20^. The questionnaire was extensively discussed with five experts from Cameroon, the Central African Republic, and Gabon. Only minor changes had to be made to improve the readability of the instrument. The questionnaire consisted of eight open-ended questions exploring respondents’ knowledge and opinion on the subject, and was included in Microsoft Excel (Supplementary file Table 1).

**Table 1:** Name and preferences to name the person who supports the pregnant woman and the new-born in maternity or antenatal consultation services

	*Name*	*Countries*											
		*Cameroon (n=17)*	*Madagascar (n=9)*	*Chad (n=7)*	*Congo, Brazzaville (n=10)*	*Guinea, Conakry (n=10)*	*Gabon (n=9)*	*Burkina Faso (n=7)*	*Central African Republic (n=10)*	*Benin (n=1)*	*Senegal (n=1)*	*Ivory Coast (n=1)*	*Togo (n=2)*
Name of the person who supports the pregnant woman and the newborn in maternity or antenatal consultation services	***Sage-femme***	15	9	7	10	10	9	7	10	1	1	1	2
	***Accoucheur***	1	0	0	0	0	0	0	0	0	0	0	0
	***Maïeuticien***	1	0	0	0	0	0	0	0	0	0	0	0
Preferences to name the person who support the pregnant woman and the newborn in maternity or antenatal consultation services	***Sage-femme***	15	9	7	10	10	9	4	9	1	1	1	2
	***Accoucheur***	2	0	0	0	0	0	1	0	0	0	0	0
	***Maïeuticien***	0	0	0	0	0	0	2	1	0	0	0	0

### Data collection

In March 2020, the online questionnaire was sent by e-mail to 140 members of the francophone professional midwifery associations in 17 French-speaking African countries. Participants were recruited by convenience sampling. E-mail addresses were available to the first author’s position in the Executive Board of the Midwifery Association of Central Africa and the Midwifery Association of Francophone Africa. Additionally, the list of member countries of the ICM was used to contact the midwifery associations. A reminder was sent out two months after the initial mailing.

Institutional approval for this study was obtained by the Executive Board of the midwifery association of Central Africa in January 2020. As this study does not involve the use of human or animal subjects or patient records or research participants’ databases (Ethical standards of the Declaration of Helsinki, World Medical Association, 2013^21^), approval was not required from a local Ethics Committee. Written informed consent was not asked, nevertheless the participants agreed to participate in the study by voluntarily filling in the questionnaire.

### Data analysis

Descriptive statistics, absolute values and percentages, were used to report the findings. The responses to the open questions were analyzed in line with the principles of thematic content analysis, with all answers coded into recurrent and common categories^22^. This involved a rigorous comparison of the answers, on paper and by means of Microsoft Excel. To avoid interpretation bias, the thematic analysis was performed independently by two coders (AHA and JV). As the next step, the categories were discussed to optimally reflect the data. Both coders organized the results and checked the final analysis to fit with the data, with significant examples of each category selected and translated from French into English. The categories are reported in order of frequency and illustrated with verbatim quotes.

## RESULTS

**Figure 1:**
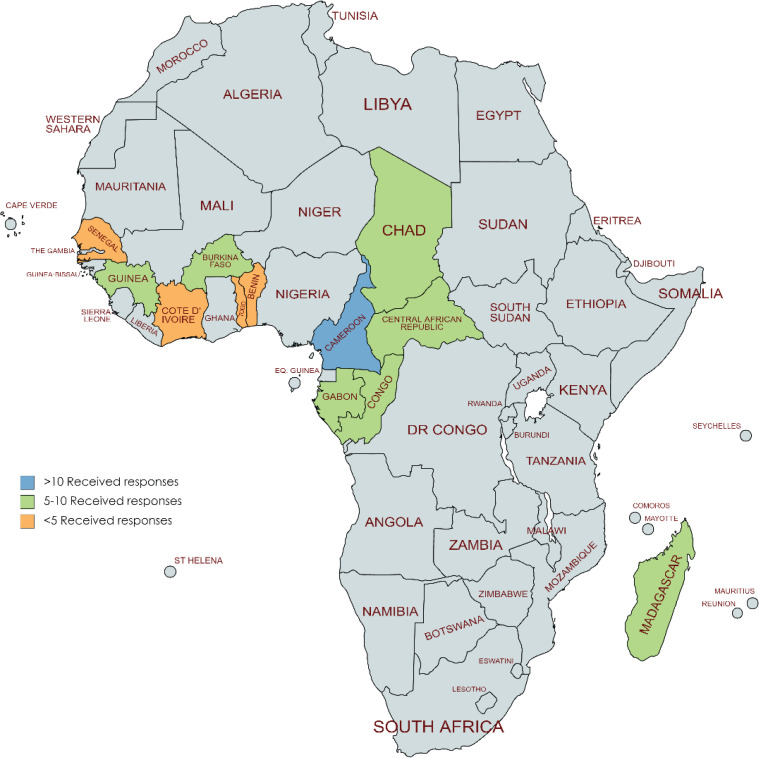
Map of responding countries

From the 140 invited midwives, 82 responses (n=58; 57%) were received. The respondents covered the Frenchspeaking African continent, meaning representation from 12 out of the 17 (n=70; 58%) invited francophone African countries, namely Cameroon (n=18), Congo (n=10), Guinea Conakry (n=10), Central African Republic (n=10), Madagascar (n=9), Gabon (n=9) Burkina Faso (n=7), and Chad (n=7). Two responses were received from Togo and one each from Ivory Coast, Senegal, and Benin (Figure 1).

We received responses from 74 females (90.2%) and 7 males (8.5%). Most respondents held the qualification *sage-femme* (n=67; 83.75%; 2 non-responses), four *maïeuticiens*, three licensed nurses-midwives (*infirmier breveté accoucheur*), and one nurse. The vast majority of respondents were aged 30–50 years (n=54; 67.5%; 2 non-responses) and most had between 0 and 20 year of experience as a midwife (n=56; 71.8%; 4 non-responses).

In nearly all responding African countries, the name of the person who provides maternity care is midwife (90%), only one responded it is an *accoucheur*. The term *accoucheur* is less and less used in today’s world while it was more used in the traditional world in the translation of the different languages of the African world to designate the one who helps to give birth (*mettre au monde*). Only one respondent coming from Cameroon mentioned the *maïeuticien* as providing maternity care in their country. While the use of the name accoucheur is disappearing and is used very little, the term *maïeuticien* is not commonly used either. Among our respondents, midwife (*sage-femme*) remains the best understood and common language in the community when referring to the main maternity care providers.

Exploring the advantages and disadvantages of the use of the terminology midwifery practice and *maïeutique*, respondents obviously prefer the words midwifery and midwife. When asking which name respondents prefer to name professionals in maternity care, the majority (95%) is in favor of midwife (*sage-femme*) (Table 1).

Four open-ended questions were available to participants if they wish to make any further comments and giving them the opportunity to share suggestions and comments about the terminology for the translation of midwifery practice and *maïeutique*. In order to complete the quantitative data, thematic content analysis identified four categories.

### Roots

Midwifery has historical, social and cultural roots and is cherished by our respondents. Additionally, it is an original name for a long time. The French translation of midwife is *sage-femme*, which refers to wisdom, *sage* means wise:

*‘Because she should be wise for women and those who take charge of women and newborns.’* (Respondent from Congo)

*‘Midwifery is wisdom, it is art.’* (Respondent from Guinea, Conakry)

Most respondents (80%) highlight the nobility of the midwifery profession.

*‘This name determines the nobility of the profession.’* (Respondent from Gabon)

*‘Midwife is related to the nobility of the practice. The word “wise” indicates competence.’* (Respondent from Togo)

*‘Reflects the nobility of the profession, known by the population.’* (Respondent from Ivory Coast)

In Africa a frequently reported disadvantage of the term *maïeutique* is that it has no cultural origin. All countries are able to translate midwifery into their traditional language, while the translation of *maïeutique* is non-existent and seems to be imposed from an elite much more than be appropriated by society and therefore difficult to understand (Table 2).

**Table 2:** Local language and English translation of midwife/maïeuticien

*Country*	*Local language*	*English translation*
Madagascar	Rasazy	Midwife
Cameroon	Mitchui/Mitchie	The person who takes the baby
	Dockita	Doctor
	Mbankui	The one that welcomes the babies
	Minga a bié	The woman who facilitates birth
	Nkuif mo	Traditional midwife
Burkina Faso	Pougroogsa	Accoucheuse or the one who facilitates birth
	Logtoré	Doctor
Chad	Djé ta doh	The person who facilitates birth
	Mara walada	The woman who facilitates birth
	Awine alnikhaba	Female midwives
	Mankodj mangué	A woman who is capable of facilitating an uncomplicated birth without difficulties
	Tawallite awine	Midwife of the women
Congo Brazzaville	Mouboutissi	A woman who facilitates birth
	Mobotsiri	She or who facilitates birth
Gabon	Emina a bialé	The woman who facilitates birth
	Mouboutissi	The one who accompanies giving birth or life
	Mbialé binenga	Midwife of the women
Guinea, Conakry	Zoole en manon	The initiate
	Sokonon nava	Traditional midwife
	Dén naminala	The one who knows the woman/the one who takes the child
	Rodjabhowo bobo	The one who takes the child/the one who knows the woman
Central African Republic	Wa mongo molengue	The person who facilitates birth
	Zotimungo molengue	The person who assists a parturient
	Wakobo	The one who helps giving birth
	Go ti mou malengue	The persons who take the children
	Nassamba	The person who facilitates birth

### Comprehensiveness

Nearly 25% of the respondents stressed that midwifery encounters gender inequity and lack of recognition. Despite the strong roots of midwifery, respondents raised institutional challenges and gender inequalities:

*‘The government does not consider us [midwives] for integration.’* (Respondent from Cameroon)

*‘No gender equity in case there are men in the job.’* (Respondent from Congo)

Referring to *maïeutique* rather than midwifery would not resolve the issue. Respondents felt that the term *maïeutique* is restrictive and does not describe the full scope of midwifery as defined by the ICM^12^. Midwifery (pratique de *sage-femme*) is more comprehensive:

*‘The term maïeutique is restrictive and just evokes ‘childbirth’, while midwifery provides a continuum of services.’* (Respondent from Togo)

But, given the confusion, about one-third of participants thought that *maïeutique* can be a means to differentiate the profession from other female health professionals.

*‘Sage-femme may be confused with any other female health professional, in our country, all female medical personnel (doctors, nurses, health workers, etc.) call them Rasazy [midwife].’* (Respondent from Madagascar)

### The midwife is acknowledged by society

The main reason that the term midwife is preferred is that the midwife is a well-known, respected and recognized person in most communities:

*‘The midwife is recognized, accepted, accountable and noble.’* (Respondent from Chad and Burkina Faso)

*‘Honesty, the source of wisdom, experience, knowledge, expertise, a professional woman competent in the pre-, peri- and postnatal art.’* (Respondent from Togo)

Moreover, the term *sage-femme* is popular in some French-speaking African countries:

*‘Popular name [sage-femme] that everyone knows.’* (Respondent from Ivory Coast)

*‘Everybody wants to be called “Rasazy” [midwife].’* (Respondent from Madagascar)

Midwife is an established name and familiar in the community and may therefore facilitate the relationship between the woman and the midwife. Conversely, the term *maïeutique* is not recognized by the professional associations and regulatory bodies in most responding countries. But most of all, the term *maïeutique* is not recognized by society:

*‘The term maïeutique is not known by public and other health professionals.’* (Respondent from Gabon).

*‘Is difficult to understand and recognize.’* (Respondent from Benin)

*‘The term does not respond to our context in Madagascar.’* (Respondent from Madagascar)

The eventual use of the term is perceived by the majority (80%) as a backlash and a loss of identity:

*‘It's like a loss of identity, for us health workers, it is easy to adapt to change but for the population, it is difficult for them to erase the current name “Rasazy” [midwife].’* (Respondent from Madagascar)

A reported disadvantage of the use of the name midwife is the potential confusion with the matrons (*matrons*), a not much respected profession in some African countries such as in the Central African Republic.

### Modernization

Respondents in favor of the terminology *maïeutique*, are typically referring to the modernization of the midwifery profession and its scientific, educational and philosophical evolution. Three-quarters of respondents from Madagascar, Cameroon and Central African Republic were in favor of *maïeutique*. In their opinion, the introduction of the term *maïeutique*, while in line with international developments, has the potential to elevate midwifery to a higher scientific level comparable with other countries. *Maïeutique* was especially identified as a way to move midwifery education in the tertiary Higher Education system, namely LMD system:

*‘Maïeutique is a branch of medicine and an art of giving birth, it expresses a hidden knowledge.’* (Respondent from Guinea, Conakry)

A minority (35%) is in favor of *maïeutique*, a midwife is not a gender-neutral designation. Few respondents (10%) think midwifery is a typically job for female only:

**Table 3:** Respondents’ opinion about the use of midwife and maïeuticien

*Country*	*Midwife*	*Maïeuticien*
Madagascar	This is the value of the profession	Modernizing the profession
	An honor	It’s like a new body
		Creating distance from the population
		Does not determine our profession
Cameroon	The one that helps give birth	Male midwife
	The attentive and wise woman	The one who helps to give birth
	The art of giving birth to spirits	The art of giving birth to ideas as in Socratic maieutics
	An independent professional in its domain	A not well-known health professional
Congo Brazzaville	Give life and protect life	Male midwife
	A profession	The ‘maïteuciens’ are called ‘infirmiers accouchers’
	A noble profession	Maïeuticien signifies male midwife
	This name is composed of the word ‘sage’ which includes: expert, skillful, saves profession of giving birth to pregnant women	
Guinea, Conakry	It is a pride, a nobility, a prestige and honor	It is a pride, a nobility, a prestige and honor
	Is wise or an expert in his art	It is a branch of medicine and an art of giving birth. It expresses a hidden know-how
	It is a wise person	The one who pulls the baby out
	Expert, skilled in his art	Male midwife
	Wise person	Who carries out the profession of midwife
Central African Republic	Name of the person responsible for the health of the mother and child	Male midwife
	Blessing, divine, trust	A male with obstetric training
Ivory Coast	The name midwife is the one that suits best to a person who does this job since the exercise, one must have certain qualities including wisdom	The name maïeuticien is better indicated because it makes it possible to distinguish between a woman and a man who exercises the profession

*‘Midwife does not include men very much, no gender equity in case there are men in profession.’* (Respondent from Ivory Coast)

*‘It is like this [midwifery] is a job for women only.’* (Respondent from Burkina-Faso)

*‘When we say midwife, it is most likely a woman but not a man.’* (Respondent from Cameroon)

## DISCUSSION

The term *maïeutique* is not used for addressing midwifery education, midwifery research or midwifery practice in most French-speaking African countries. The added value of the use of *maïeutique* is uncertain and respondents thought that change can call into question the recognition and identity of the profession. The term *sage-femme* is comprehensive and clear for members of society. Likewise, the term midwifery is representative and significant for the profession and correspondents with international terminology used by ICM. Imbedded in historical, social and cultural roots, the term *sage-femme* is rich in its meaning. More especially, the term appeals to wisdom that appears to be very significant to our respondents, and as a consequence they do not want to give up the term *sage-femme*. Even when the term *sage-femme* is not gender neutral, the term appears to be accepted by male midwives. An appropriate terminology of the term midwifery in French could generate added value in the community and by other professionals. This makes it possible to distinguish the midwifery profession from other maternity care professionals. However, the wish of respondents to solve the mentioned confusion with the term *maïeutique* is hardly believable. A response more credible is to find it in the Bill of Rights for Women and Midwives^23^ when approaching governments and demanding change to improve midwifery and maternity services.

In the light of the French literature on the subject^6^ and present survey, it seems likely that the terms midwifery and *maïeutique* are perceived as not interchangeable. Most of our respondents emphasized a potential significant loss of meaning related to their discipline when using *maïeutique*. The findings from our study are in accordance with the findings of Meyer et al.^6^ who investigated the perception of the term *maïeutique* and concluded that the terms *maïeuticien* and *maïeutique* gained little acceptance by midwives from French-speaking countries all over the globe.

If midwifery is desired by the majority of the respondents, it remains unclear what name is better suited for the profession within the upcoming LMD system in Africa. Making midwifery related higher education accessible in Africa, provides a pathway by which to increase availability of skilled midwives trained to ICM standards^24^. The integration of midwifery programs of study within degree-granting pathways, such as the LMD level, has been perceived in Europe^25^, some Africa and Middle-East countries^14^ as a reform that offers midwives the opportunity of academic advancement and may pave the way to career progression. Nevertheless, it has to be determined which terminology is needed to distinguish the different educational levels.

The upcoming LMD educational reform in Africa is comparable with the Bachelor’s, Master’s and doctorate system in Europe and in Canada. However, worldwide midwifery education has undergone a number of reforms in the past few decades. In several countries, it has shifted from vocational training to academic education^26^. The higher education reform, known as the ‘Bologna process’ aimed to create convergence in higher education among a number of European countries and enhance opportunities for mobility, employment, and collaborative research. It also indicated a transparent and easily compared system of academic degrees, generating a new educational system in three cycles^27^.

In many countries, the principles of the Bologna process have been realized, although to varying degrees throughout Europe^28^. Many midwifery education programs are currently provided at Bachelor’s level in some countries, however midwifery education is still offered as a vocational or an apprenticeship model^29,30^, though in Europe the discussion of distinguishing midwives in relation to their educational level was not on the agenda until the translation of midwifery into *maïeutique*.

According to Nguyen^4^, we can expect difficulties when not using the term *maïeutique* to name midwifery research^4^. Moreover, for the French National Conference of Maïeutics’ Teachers, research in the field of *maïeutique*, inextricably interconnected with medical sciences, in this case *maïeutique* goes far beyond research on midwifery practices only^31^. Conversely, the French version of the State of the World’s Midwifery (co-authored by United Nations Population Fund, ICM and World Health Organization) does not use the term *maïeutique* either for midwifery education or midwifery research. This is similar as in other countries, in Switzerland recently, the term *la pratique avancée sage-femme* (Advanced Midwifery Practice) is introduced to name an accredited practicing midwife with a Master’s degree, in-depth expertise, research skills, and advanced leadership competence^32^. Further research is warranted to identify which term is best suited to determine midwifery education and midwifery research.

The midwives from the midwifery association of Central Africa and the midwifery association of francophone Africa proposes to distinguish the different education levels of midwives as: *‘licence en pratique (science) sage-femme’, ‘master en pratique sage-femme’* and *‘doctorat en pratique sage-femme’*. They clearly advocate the preservation of the term midwifery practice instead of *maïeutique*. The proposal of the midwifery associations of Central Africa and francophone Africa is undoubtedly confirmed by the results of our study. Moreover, the French version of the State of the World’s Midwifery does not contain the terms *maïeutique*/ *maïeuticien* at all, but the term midwifery practice (*l’état de la pratique de sage-femme*) is used^33^.

It is identified that in low- and middle-income countries there is a lack of shared understanding about what midwifery is, and the level of education, training, support and regulation that is required to enable women and newborns to receive quality midwifery care. This lack of understanding of midwifery may explain the perception of the role as essentially ‘women's work’ and why gender inequality is described as the primary barrier to the advancement of the midwifery profession^34^. While in Central Africa several countries integrated male midwives in their health system, only in 4 of 11 responding countries (36%) men can access midwifery education, namely in Cameroon, Burkina Faso, Chad and the Central African Republic. However, from those countries, and their responding male midwives, there is no call to strongly consider the introduction of the terms *maïeutique* and *maïeuticien*. Additionally, in the English terms of midwife and midwifery there is no change of professional name depending on the gender of the health professional.

### Strengths and limitations

This is the first study to explore the opinion of francophone African maternity care professionals regard to the terms: midwifery, *sage-femme*, and *maïeutique*. This study was limited to midwives from 17 African countries, all members of the midwifery association of francophone Africa. As we did not include North African francophone countries such as Tunisia, Algeria and Morocco, the results of our study are not generalizable to all francophone African countries. To identify the worldwide impact of the terminology of *maïeutique*, a replication of this study including midwives from all francophone countries in the world is warranted.

We received responses from 7 male midwives which is 8.5% of all respondents. Worldwide, the proportion of male midwives is between 0% and 50% and dependent on social context in countries. In 2019, the proportion of male midwives in responding countries was between 0% (Guinea Conakry, Ivory Coast and Benin) and 13% (Burkina Faso)^35^. Despite the adequate representation of male midwives in our study, more research is warranted to explore their opinion on the use of the terminologies *pratique de sage-femme/maïeutique* and *sage-femme/maïeuticien*.

As illustrated by some respondents and as a study in Benin and Burkina Faso thematizes^8^, midwifery cannot ignore power relations and the instability of professional status. These difficulties do not necessarily mean that the solution lies in a change of name. Priority must be given to the work of professionalizing midwives and their profession, so that they can better assert their skills and be better recognized by society and other maternity care professionals.

## CONCLUSIONS

*Maïeutique* is restrictive, not specific, abstract and unknown by the public and other health professionals. However, a minority of our respondents are of the opinion that *maïeutique* needs some further consideration as the term has the potential to differentiate midwives form other maternity care professionals and can diminishing role ambiguity, value midwifery practice, and modernize the midwifery identity.

Our respondents are open to reflection about the evolution and modernization of terminology, but there is no consensus. Internationally, midwives are following developments on the linguistic subject of midwifery versus *maïeutique* but to date the discussion of terminology of our distinguished profession is not high on the agenda in francophone Africa. If the discussion is to be held, it is essential that midwives themselves are actively involved. This need to be prepared by extensive discussions with midwives in the French-speaking countries over the world, also or most important stakeholders, women, need to be involved. Nevertheless, at all times we need to be cautious not to break away from midwives’ cherished historical, social, and cultural roots.

## Data Availability

The data supporting this research are available from the authors on reasonable request.
